# Strontium and Magnesium in Otoliths Can Trace *Schizothorax grahami* (Regan, 1904) Life History

**DOI:** 10.3390/ani15213170

**Published:** 2025-10-31

**Authors:** Yang Zhou, Zhongtang He, Weijie Cui, Qun Lu, Jianguang Qin, Zhaofang Han, Jianhu Liu, Tao He

**Affiliations:** 1 College of Fisheries, Southwest University, Chongqing 400715, China; swu1997zy@163.com (Y.Z.); hzt944581429@163.com (Z.H.); cuiweijie2022@163.com (W.C.); swluqun2023@163.com (Q.L.); zhaofanghan@swu.edu.cn (Z.H.); 2 College of Science and Engineering, Flinders University, Adelaide 5001, Australia; jian.qin@flinders.edu.au; 3 Key Laboratory of Freshwater Fish Reproduction and Development (Ministry of Education), Key Laboratory of Aquatic Science of Chongqing, Chongqing 400715, China

**Keywords:** strontium, magnesium, otolith, *Schizothorax grahami*, life-history

## Abstract

This research examines the otolith microchemical characteristics of the critically endangered kunming snout trout (*Schizothorax grahami*) collected from the sources section endemic to the Chishui River, China, a tributary of the upper Yangtze River, and the metal concentrations in the water of fish habitats. Among the analyzed elements, strontium (Sr) exhibited consistent distribution patterns across otolith rings, as observed through face-scan imagery (scanning the polished surface of the otolith cut), with a relatively low coefficient of variation. Statistical analysis using a paired two-tailed *t*-test revealed significant differences in Sr:Ca ratios among samples from the three river sections. Notably, magnesium (Mg) levels in the otolith core during the early life stages of the fish were notably higher compared to non-core regions. Similarly, Mg concentrations of water were elevated in the spawning grounds relative to non-spawning areas. This suggests that otolith Mg content may be influenced by the specific water conditions of the spawning grounds. Furthermore, Mg:Ca ratios in the otolith core displayed significant variation among samples from U3, L2, and other sites, implying that the fish in these samples originated from distinct spawning locations. These findings demonstrate that strontium and magnesium in otoliths can serve as effective markers for reconstructing the life history of *S. grahami* in the Chishui River and can contribute to the management of different fish stocks.

## Simple Summary

Life history has always been a key research focus for fish conservationists. However, the connection between life history and the environment has not yet been fully established. By studying the microchemical information of otoliths in *Schizothorax grahami* from the source section of the Chishui River in China, we found that only strontium (Sr) is regularly distributed in the otolith ring. The values of Sr:Ca and the change frequency significantly differ among the three reaches (intra- and between-class). Mg content in the otoliths is correlated with the water environment of the spawning ground. Meanwhile, the values of Mg:Ca in the otolith core showed significant differences among the samples, indicating that those samples came from different spawning grounds at the early stage of their life history. And, through the characteristics of Mg in the otoliths, the source and life history of *S. grahami* in the Chishui River could be identified. Therefore, strontium and magnesium in otoliths could be used to trace the life history of *S. grahami* in the Chishui River.

## 1. Introduction

In fishery research, otoliths are a key tool for determining fish age and have great practical value in the study of fish population structure and life history [1,2]. The process of otolith formation is mainly affected by genetic and environmental factors. The variation of environmental changes can affect fish physiology, resulting in changes in otolith morphology, elemental composition [3,4,5,6,7], and population specificity [8]. Otolith morphology and chemical composition have been used to distinguish fish stocks from different locations [9]. During fish growth, chemical elements in the water are constantly incorporated into the otolith matrix [10], effectively recording the environmental characteristics in the whole life history [11,12,13]. The otolithic rings may produce corresponding changes when fish undergo metamorphosis, environmental changes, and external stress [14]. Hence, the microchemistry of fish otoliths serves to identify spawning, feeding, baiting, overwintering habitats, and migratory channels.

Recent research in fisheries has enhanced the understanding of otoliths microchemistry. The multi-point laser ablation sampling (LA-ICPMS) technique can successfully recognize the Sr:Ca and Ba:Ca distribution in the otolith core area by analyzing tapertail anchovy (*Coilia nasus*) populations on China’s offshore coast. The success rate of population discrimination obtained was 72%, indicating that the otolith elements have a strong identification ability [15]. In stock discrimination, anadromous fish exhibited significantly higher Sr:Ca concentrations in their otolith primordia than resident fish [16]. Measurements of Sr:Ca values of the otolith, combined with the literature and reports from previous surveys, spawning grounds, hatcheries, and migration routes of *Electrona carlsbergi* were identified [17]. Based on the ratio of Sr:Ca content in the otolith core area, it is hypothesized that the spawning ground of *Liza haematocheila* in the Yangtze River estuary is located in intertidal freshwater and semi-saline waters [18]. Wang et al. used the LA-ICPMS technique to measure elements in the otoliths of *Hexagrammos otakii* in the Bohai and Yellow Seas. The ratio of trace elements to calcium in an otolith can distinguish different *Hexagrammos otakii* stocks [19]. The above case studies illustrate the effectiveness of otolith microchemistry in delineating fish stocks and life history analysis. To date, otolith microchemistry has been studied primarily on marine fishes, especially regarding the differentiation of life history types and their association with habitats. However, research on freshwater fish species in China has yet to be fully explored, with current investigations remaining relatively limited in scope and depth.

As *Schizothorax grahami* (Kunming snout trout) is a dominant species in the resource section of the Chishui River, and since it is critically endangered and endemic to the Yangtze watershed, especially the Chishui River tributary in the mountainous upper section, studies have focused on physiology and biochemistry [20,21,22], reproduction [23], molecular biology [24], and diseases [25], while in-depth exploration of its otolith information has not been reported. Within this study, the otolith microchemical characteristics were used to distinguish the life history types of *S. grahami* and test the relevance of otolith information and habitat environment in the Chishui River. The results of this study provide a better understanding of the life-history and stock characteristic of *S. grahami* in the basin. The approach of this study lays a theoretical and practical foundation for studying life history and evaluating the distribution of fish stocks after release into the wild for stock enhancement.

## 2. Material and Methods

### 2.1. Study Area and Field Sampling

**Figure 1:**
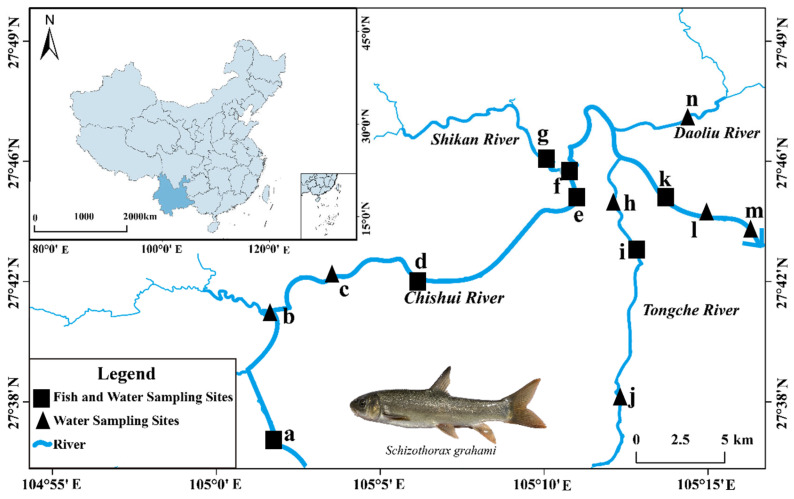
*S. grahami* and water sampling locations in Chishui River, March 2022. (The arrow points in the direction of water flow).

A total of 97 adult fish samples were captured from 13 sites in the source section of the Chuishui River in March 2022 with cage traps and cast nets. After measuring the total length (TL, 0.1 mm) and total weight (W, 0.1 g) of each specimen in the field (Table 1), we extracted lapillus otoliths (left and right) from the inner ear of all specimens with fine forceps in the laboratory. Following the collection process, the samples were cleaned using an ultrasonic cleaning machine (YM-008S) to eliminate any residual membranes or surface contaminants. After drying, they were stored in appropriately labeled plastic tubes for subsequent analysis. Sampling stations for *S. grahami* were established and water sampling was conducted in the Chishui River, Shikan River, Daolin River, and Tongche River, China. The samples from Yudong (a in Figure 1) were designated as the upstream reaches, as the flow reduction observed in this area was attributed to the demolition of the power station located between Yudong and the other sampling sites. This disruption significantly impacted the fish migration route due to the accumulation of debris on the riverbed. Meanwhile, the samples from h-m (in Figure 1) were classified as the downstream reaches, given the confluence of the Daoliu River and the Tongche River, which modified the downstream habitats. The remaining samples were categorized as the midstream reaches. In this river, Shikan, Daolin, and Tongche are tributaries of the Chishui River, which is a primary tributary of the Yangtze River.

### 2.2. Otolith Microchemistry Measurements

Three samples were randomly selected for microchemical analysis among those collected from each reach (The samples from the upper reach were numbered U1, U2, and U3, the samples from the middle reach were numbered M1, M2, and M3, and the samples from the lower reach were numbered L1, L2, and L3). After otoliths were washed and dried, they were fixed on slides with neutral resin and polished with sandpaper of 1000#, 3000#, and 5000# grit. Until the rings and core were clearly observed under the microscope, they were analyzed by laser ablation (Analyte HE, CETAC, Omaha, NE, USA) (LA) inductively coupled with a plasma mass spectrometer (NexIon 1000G, PerkinElmer Nexlon, Waltham, MA, USA) (ICPMS). The common elements (Li, Na, Mg, Si, S, K, Ca, V, Mn, Fe, Cu, Zn, Pb, Hg, Ba, Se and Sr) were selected for measurement based upon the local geology. The center of the otolith core area was selected, and samples were taken at 5 µm intervals to the edge closest to the core in sequence. Because variation in otolith size caused the number of scanning points to change, the elemental content of 344 scanning points was used to determine the smallest otolith, which dictated the length of the scan used for quantitative analysis. Each variation of the element coefficient was calculated, and the chemical species with the smaller coefficient of variations was selected as the main elements for analysis.

The following is the formula for the elemental coefficient of variation:

$$CV=\frac{S}{M}\times 100$$

*S*—Standard deviation of elemental content at scanning points.
*M*—Mean value of elemental content at scanning points.

### 2.3. Metal Element Measurements in the Water Environment

The water samples were collected in 100 mL increments at each sampling site (Figure 1), and the site information is shown in Supplementary Table S2. The water samples were filtered by a 0.45 μm pore size filter membrane in the laboratory. The metal elements in the water were measured by an inductively coupled plasma mass spectrometer (Agilent 7900, Agilent, USA) in the Southwest University Analytical Testing Center. Ca, Na, K, Sr, and Ba, the five metallic elements with small coefficients of variation, and Mg, the companion element of Ca, were chosen for analysis.

### 2.4. Statistical Analysis

Data statistics and first-order differences were conducted using Microsoft Excel 2021. SPSS 26.0 was used for one-way ANOVA and correlation statistical analyses. Origin 2022 was employed for plotting. The *p*-value was set at 0.05 for significant differences.

### 2.5. Ethics Statement

All protocols related to experiments and fish collections in this study were approved by the Regulation on Animal Experimentation at Southwest University. (No. swu-20220225219).

## 3. Results

### 3.1. Characteristics of Sr in S. grahami Otolith

**Figure 2:**
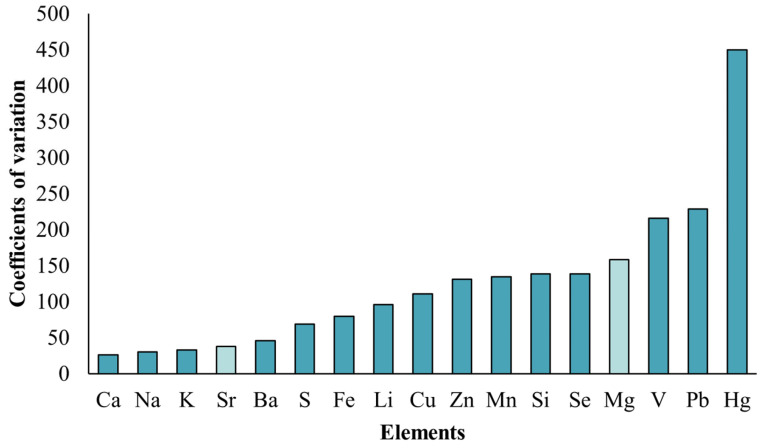
Coefficients of variation of 17 elements in lapillus otoliths from *S. grahami* sampled from the Chishui River in March 2022 (primary research elements in light color).

**Figure 3:**
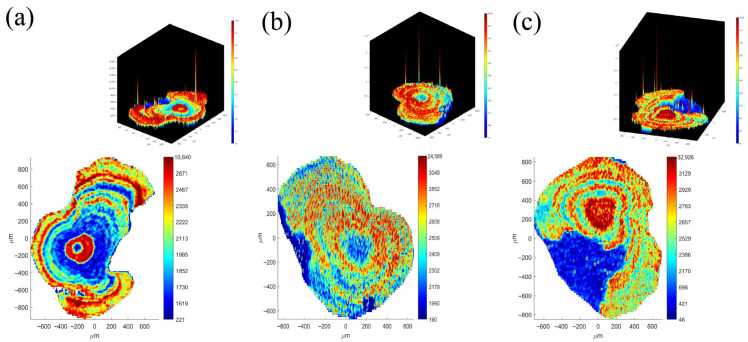
Microchemical map of elements in the lapillus otolith from *S. grahami* sampled from the Chishui River in March 2022, and microchemical three-dimensional map of Sr in (**a**) Upper reaches, (**b**) Middle reaches, and (**c**) Lower reaches. Vertical coordinate on the right side = cumulative distribution; the unit is 10^−6^ g/g. Microchemical maps of other elements in Figure S2.

In the microchemical analysis for all otoliths, the coefficients of variation (CV) of all elements was calculated, and the major five elements were Ca, Na, K, Sr, and Ba (Figure 2). Combining the elemental content in otoliths with the otolith face-scan images (Figure 3 and Figure S1) and the spatial heterogeneity of environmental chemical elements, Sr, Ca and Mg (associated elements of Ca) were selected as the primary elements in this study. Moreover, only the Sr element was regularly distributed in the otolith ring with the growth of the otolith (Figure 3).

To compare the metal element contents from the three reach groups, the mean values of Sr:Ca for each group were first calculated to examine their distribution pattern. The asymptotic difference of all three reaches was not significant (*p* > 0.05) (Supplementary Figure S2). Combined with the normal Q–Q plots of the three reaches (Supplementary Figure S3), it is clear that the above data consistently conformed to a normal distribution, allowing for paired dichotomous testing of the means. The results of the paired *t*-test of the mean (Table 2) showed significant differences among each pair in terms of microchemistry.

**Figure 4:**
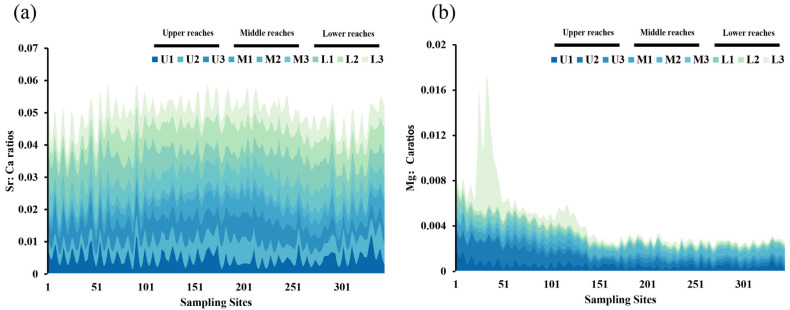
Fold line chart of the ratio of Sr:Ca (**a**) and Mg:Ca (**b**) of *S. grahami*. U1–U3 represents three samples from the upper reach; M1–M3 represent three samples from the middle reach; L1–L3 represent three samples from the lower reach. Sampling Sites refers to the sequential number of the laser ablation spot, where each spot has a diameter of 5 μm. (The definitions provided here apply to all subsequent uses in the text).

The trends of the ratios of the five elements Sr, Ba, Mg, Na, and K to Ca through the otolith face scan, collected from the upper, middle, and lower reaches, showed different variation characteristics of *S. grahami* otoliths in Figure 4 and Figure S3.

**Figure 5:**
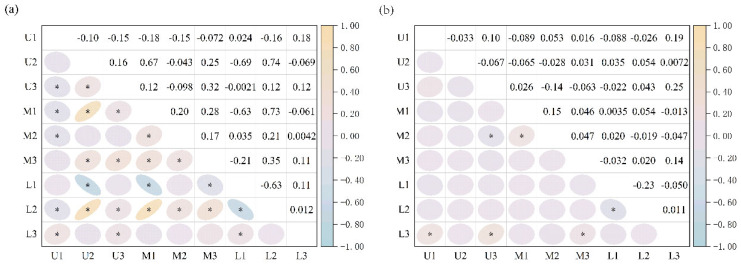
The correlation test of otoliths microchemistry line scan (**a**). Data has not been processed. (**b**). After first-order difference processing. (“*” denotes *p*-value < 0.05).

As for the frequency of Sr:Ca ratio variations in a pointwise line chart, lower reaches L1 and L2 changed more gently (minor fluctuations in values), and the rest of the samples exhibited a higher frequency of high-frequency oscillations. From the changing trend of Sr:Ca values, upper reaches U1 and U2 had an increasing trend after the 300th scanning point, while U3, M3, L1 and L3 had a decreasing trend (Figure 4a). There was a slow increase in the Mg:Ca values in the first 40 scans, followed by a sharp increase that reached its maximum at 42 scans (Figure 4b). Pearson’s correlation test was performed on the line-scan results of the nine samples to analyze the correlation among each sample in detail. The correlation among the nine samples was complicated, and no conclusive pattern emerged (Figure 5a). To reduce the irregular fluctuation of the data, Pearson’s correlation test was performed again after the data underwent the first-order differencing process (Figure 5b). U1 was significantly correlated only with L3, U3 was significantly correlated with M2 and L3, and U2 was non-significantly correlated with the other samples. Intraclass correlation was identified between M1 and M2 and L1 and L2. The results of the correlation analysis generally corresponded better to the changes in the fold line chart.

### 3.2. Characteristics of Mg in S. grahami Otolith

**Figure 6:**
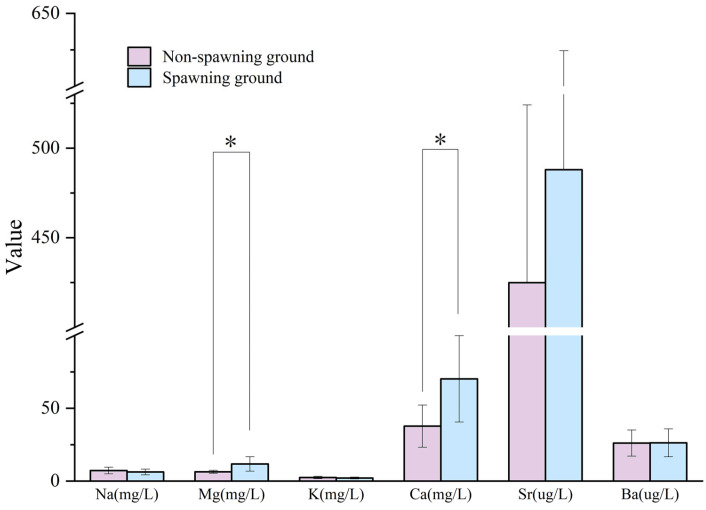
*T*-test (one tail) for the content of six metals in the water of spawning grounds (including sampling points within 1 km of the river reach of spawning grounds) and non-spawning grounds from the Chishui River in March 2022. (“*” denotes *p*-value < 0.05).

**Figure 7:**
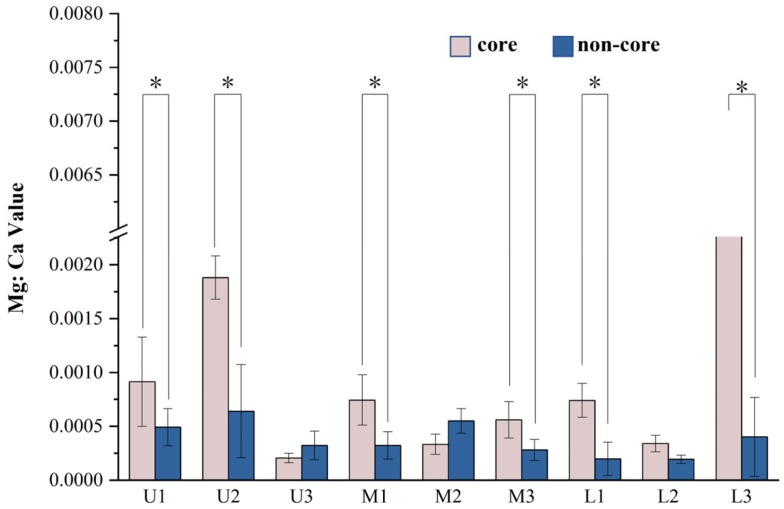
The values of Mg:Ca *t*-test in core and non-core areas of nine samples of *S. grahami* sampled from the Chishui River in March 2022. (“*” denotes *p*-value < 0.05).

Most of the Mg:Ca values in the otoliths were significantly higher in the core area than in other areas, as shown in Figure 4. Furthermore, Ca was significantly higher in the water of the spawning ground than in the non-spawning ground, and the average content of Mg in the water of the spawning ground was nearly twice that of the non-spawning ground (Figure 6). A *t*-test of the Mg:Ca values between the core areas (the first 50 scanning points of the otolith profile) and the non-core areas (the 51st to the 344th scanning point of the otolith profile) indicated that the Mg:Ca values of the core areas are significantly higher than those of the non-core areas, except in the U3, M2, and L2 samples (Figure 7).

## 4. Discussion

### 4.1. Characteristics Analysis of Sr in S. grahami Otolith

The determination of chemical elements in otoliths is not only important for reshaping the life history analysis of the fish but also allows for stock delineation. Fish stocks have different life histories and environments, resulting in variations in the level of chemical elements accumulated on otoliths [26,27]. In this work, elemental surface scans of otoliths from *S. grahami* revealed the highest Ca content. This observation is in agreement with the findings for *Ommastrephes bartramii* [28], *Oncorhynchus keta* [29], and *Electrona carlsbergi* [17]. The remaining elements with higher content were Na, Sr, K and Si, respectively, which is different from the measurements of other scholars, probably because the chemical elements in otoliths are influenced by various factors such as water, geology, and physiological and biochemical activities of fish species.

In this study, we analyzed the differences in the Sr:Ca values of each scanning point in the otolith through the paired two-tailed *t*-test. There were significant differences among the same otolith points in the three groups. This indicates that *S. grahami* from the three reaches have different life histories. The otolith microchemistry of four fish species in the Yangtze River estuary was studied [30], and four species showed different life histories, similar to the result of the present study. The otolith microchemistry of *Coilioa Nasus* was analyzed in three river sections (Changshu, Jingjiang, and Nanjing) of the Yangtze River [31], showing that *C. Nasus* is both migratory and freshwater sedentary with a mixed habitat. Another study on five different populations of *C. Nasus* in different water areas found that Sr/Ca and Ba/Ca values can distinguish fish stocks [32]. Jing et al. measured the chemical elements in the otoliths of juvenile silver carp taken from two water areas at three time periods. They found that Si/Ca and Sr/Ca values significantly differed in the otoliths of juvenile silver carp from both water areas and could determine the juvenile silver carp population in the two water areas [33]. The chemical elements in otoliths of juvenile *Larimichthys polyactis* from the Yellow Sea and the Bohai Sea have been analyzed, and there are significant differences in the chemical element contents on the otoliths of *L. polyactis* from the two water areas, which means they could be classified into two geographical populations [34].

Line scans of the nine samples in this study showed various trends. After first-order difference processing, the correlation test showed that the Sr:Ca ratio fluctuations in otoliths were more consistent with the line plots and exhibited significant differences among the three reaches (within and between classes). There was no significant correlation between U2 and all samples in the basin, indicating that U2 may come from other stocks, as there are multiple stocks in the upstream reaches. U3 has a significant correlation with L3, and the significance is the highest in this analysis. However, they do not belong to the same reach. Since there are many large drops of water flow in the river basin, and since *S. grahami* do not have swimming and jumping ability like salmon [35], the probability of high-frequency gene exchange between the upper reaches and lower reaches is low. L1 and L3 are significantly negatively correlated, and the correlation coefficient is the lowest in the negative correlation. Otoliths signals suggest the potential presence of stocked individuals. In combination with the local situation in the rivers, the significant correlation of Sr:Ca values in the groups between reaches may be caused by fish proliferation and artificial release. The research region is a national nature conservation reserve. Fish proliferation and release activities have been carried out from 2010 to 2020, and 200,000 *S. grahami* were released in September 2020 to protect the river’s biodiversity. Therefore, the *S. grahami* population in the Chishui River comprises released and wild stock. Zhang Y et al. [36] compared the otolith microchemistry of released stock and the wild stock of black seabream. These two populations had significant differences in the coefficients of variation for the Ba:Ca ratio, which is similar to the results of the present study, but the differences between the two black seabream populations were not reflected in the curve of the line scan results, possibly due to the difference in geographic environments and fish species.

Meanwhile, the correlation analysis between the Sr:Ca value and the Sr:Ca value in the line graph indicates that multiple stocks may also exist in the same geographic area, as these stocks have a strong relationship. Zhou et al. used otolith profiles to study *S. grahami* stock in the river source section of the Chishui River, and the comprehensive discrimination success rate was 59.7%, and there are multiple *S. grahami* stocks in the river, which is in line with the present study [37]. Chen studied the otolith microchemical characteristics of knife crabs (*Naja nasutus*) from the Jiangsu section of the Yangtze River in the three sections at Changshu, Jingjiang, and Nanjing, and found that *C. nasus* was both migratory and freshwater sedentary [31]. These results suggest that otolith microchemistry can be used to distinguish the life history of the crabs, which is consistent with the findings of the present study. More than three stocks of *S. grahami* were present in the source section of the Chishui River, and these stocks consisted of the released stock and wild stock, indicating their mixture of life history.

### 4.2. Characteristics Analysis of Mg in S. grahami Otolith

The water environment affects the otolith microchemical element content and distribution. Therefore, the microchemical characteristics of otolith should reflect information about the whole life history of the fish [37,38]. Microchemical analysis of otoliths is commonly employed to differentiate fish stocks within a species, providing insights into habitat-specific or geography-related differences in their origins [39,40]. In this study, by analyzing the Mg:Ca values in the core and non-core zones of otolith profiles, we found that the Mg element in the core zone of seven samples was higher than that in the non-core zone, indicating the fish come from different spawning grounds because the elemental content in the ambient water will affect the elements deposited in otoliths [7]. This suggests that the higher Mg content in the core zone of *S. grahami* otolith is probably due to the higher Mg content in the environment of the larval and juvenile periods. In addition, examination of metal concentrations across 14 distinct habitats demonstrated that magnesium and calcium levels were significantly elevated in spawning ground waters relative to non-spawning areas.

Otoliths begin to form in the embryonic stage of fish devlopment, and *S. grahami* produce sticky eggs attached to the riverbed substrate. During incubation, metal elements in the water column can enter the embryo through diffusion [41]. After hatching, the fry does not have strong swimming ability. At this time, the fry will stay in the slow-water-moving area. During this period, the metal elements in the water body enter the fish body through feeding and respiration and then enter and are deposited in the otolith [42] Hence, higher levels of metal elements in the spawning grounds and nearby water bodies will be deposited in the otoliths. This phenomenon also occurs in marine fishes such as *Sillago robusta* [43], *Thunnus thynnus* [44], *Sparus microcephalus* [36] and others. Hence, U3, M2, and L2 do not exist in the spawning ground of this survey in the early stage.

Combined with the correlation test results for Sr:Ca values, it is highly likely that these two samples belong to released populations and came from different farms. Although the Mg:Ca values in the L3 core area are significantly higher than those in the non-core area, there is a slow increase in the Mg:Ca values in the first 40 scans, with a sharp increase in the 42 scans. Based on the above inference, it is possible that L3 also belongs to the released populations via stock enhancement.

In summary, U3, M2, L2, and L3 may be augmented release populations, but this is less certain in M2 and L3, and the remaining samples may be the wild populations. However, this needs further verification, as no experiments related to the changes of microchemical elements in the otoliths of cultured fishes were designed in this study. Through the examination of Mg in otoliths, the life history patterns of *S. grahami* of different stocks might differ in the Chishui River.

## 5. Conclusions

In this study, we investigated the microchemistry of *S. grahami* otoliths from the upper, middle, and lower reaches of the Chishui River. Only Sr is regularly distributed in the otolith ring. The values of Sr:Ca and the change frequency significantly differ among the three reaches (intra- and between-class). Therefore, through the characteristic of Sr in otolith, the *S. grahami* from the three reaches may belong to different stocks, indicating their life history is different. The otoliths core, formed during the early life stages of *S. grahami*, exhibits significantly higher Mg levels compared to non-core regions. These elevated Mg concentrations closely align with those observed in the spawning ground water environment, which are notably more significant than in non-spawning areas. This similarity suggests that Mg levels in the otoliths are influenced by the water conditions of the spawning grounds. Furthermore, significant variations in the Mg:Ca ratio within the otolith core imply that the analyzed samples likely originated from distinct spawning grounds during their early life stages. By examining both Sr and Mg in the otoliths, researchers can effectively trace the life history and determine the germplasm resources of *S. grahami* in the Chishui River. Finally, while our descriptive analysis successfully identified significant microchemical heterogeneity corresponding to environmental factors across the Chishui River, a critical next step involves refining these findings for fisheries management applications. Future research, leveraging comprehensive water chemistry baseline data across all putative habitats, should focus on applying advanced multivariate classification methods, such as discriminant function analysis (DFA) or Bayesian assignment models, to accurately predict the natal origin of individual *S. grahami*. This will provide the predictive power necessary for quantifying stock connectivity and improving conservation strategies.

## Figures and Tables

**Table 1:** Basic information about the sampling sites in different rivers.

Reaches	Sampling Sites Number	Number	Total Length Ranges (mm)	Weight (g)
Upper	a	34	135–212	39.8–339.4
Middle	b–g, n	33	82–247	11.6–383.6
Lower	h–m	30	113–238	23.2–328.5
Total	97			

Note: The sampling point number in the table corresponds to Figure 1.

**Table 2:** The results of paired *t*-test of the mean (df: 343).

Paired Ablation		t	*p*-Value
Pairing 1	upper-middle	22.079	0.00001
Pairing 2	upper-lower	−20.013	0.00001
Pairing 3	middle-lower	−47.026	0.00001

## Data Availability

The data of the present study are available from the authors upon reasonable request.

## Supplementary Materials

The following supporting information can be downloaded at https://www.mdpi.com/article/10.3390/ani15213170/s1. Supplementary Figure S1: Microchemical map of elements in otolith (a) Upper reaches (b) Middle reaches (c) Lower reaches; Supplementary Figure S2: Normal Q–Q diagrams for the three groups. (a) Upper reaches (b) Middle reaches (c) Lower reaches; Supplementary Figure S3: Point-fold line chart of the ratio of 3 elements (Ba, K, Na) to Ca of *S. grahami*. Supplementary Table S1: One-sample K–S test for three groups; Supplementary Table S2: Water sampling sites information table.
