# Supplementary material for: Strontium and Magnesium in Otoliths Can Trace Schizothorax grahami (Regan, 1904) Life History
Source: Animals (Basel). 2025 Oct 31;15(21):3170. doi: 10.3390/ani15213170 (PMC12611025; doi:10.3390/ani15213170)
Supplement: Supplementary file 1 [file animals-15-03170-s001.zip › animals-3906315-supplementary.pdf]

animals

Supplementary Information: Strontium and Magnesium in Otoliths Can Trace *Schizothorax grahami* (Regan, 1904) Life History

Yang Zhou [1], Zhongtang He [1], Weijie Cui [1], Qun Lu [1], Jianguang Qin [2], Zhaofang Han [1,3], Jianhu Liu [1,3,*] and Tao He [1,3,*]

[1] College of Fisheries, Southwest University, Chongqing 400715, China; swu1997zy@163.com (Y.Z.); hzt944581429@163.com (Z.H.); cuiweijie2022@163.com (W.C.); swluqun2023@163.com (Q.L.); zhaofanghan@swu.edu.cn (Z.H.)
[2] College of Science and Engineering, Flinders University, Adelaide 5001, Australia; jian.qin@flinders.edu.au
[3] Key Laboratory of Freshwater Fish Reproduction and Development (Ministry of Education), Key Laboratory of Aquatic Science of Chongqing, Chongqing 400715, China
* Correspondence: liujianhu@swu.edu.cn (J.L.); hh1985@swu.edu.cn (T.H.); Tel.: +86-3594621973 (J.L.); +86-5023177126 (T.H.)

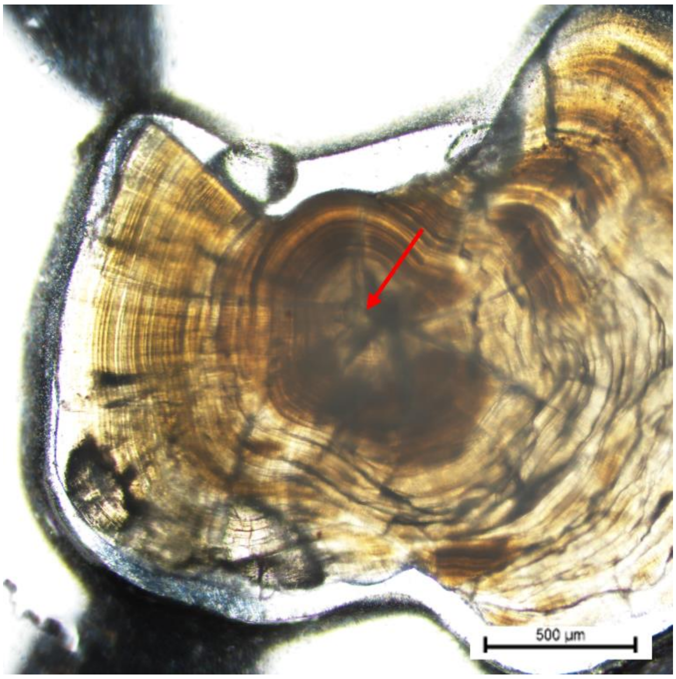

Figure S1. The photo after the otolith has been ground, *s. grahami* sampled from the chishui river in march 2022 (the area pointed by the red arrow is the core area).

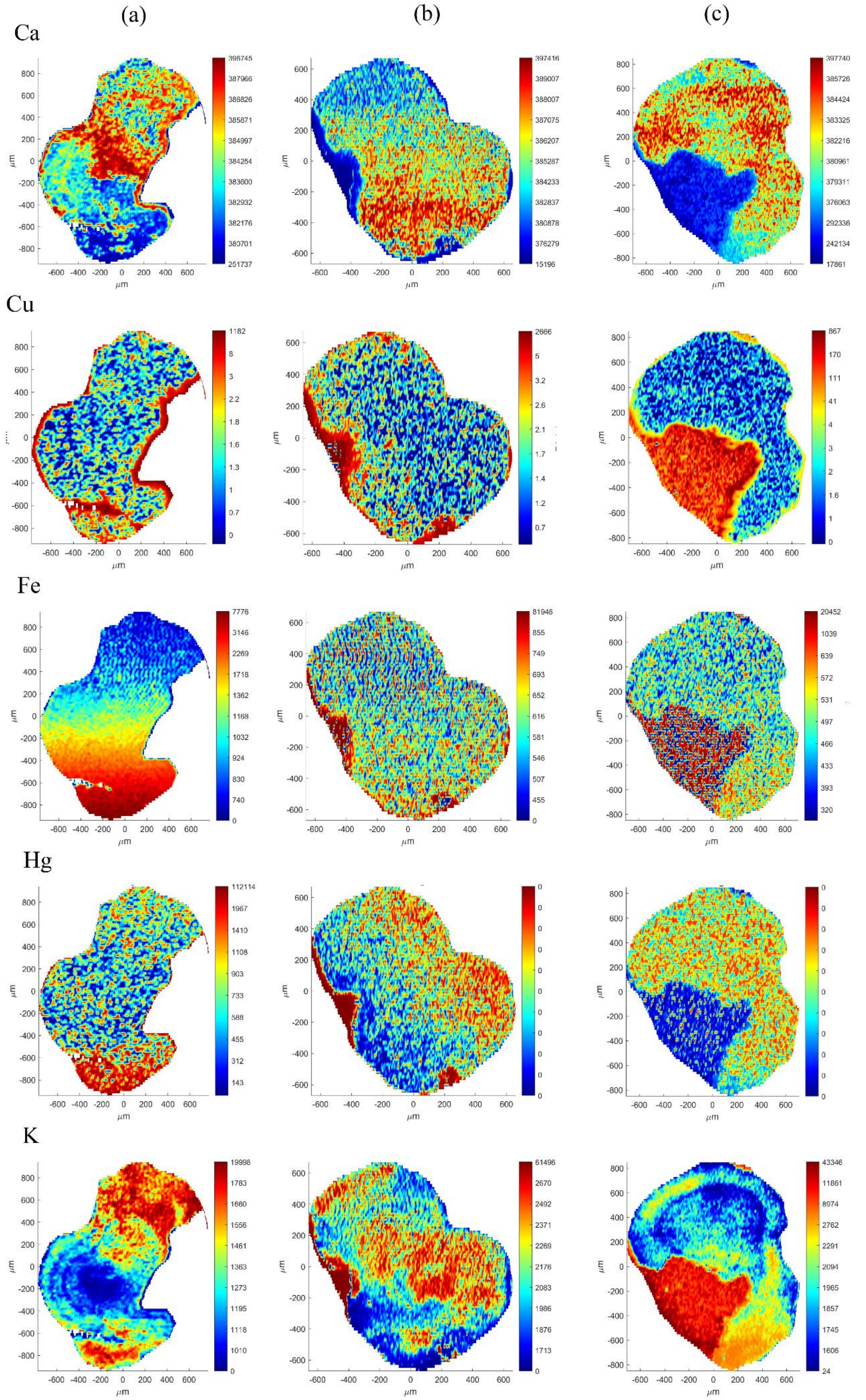
(a)
(b)
(c)
Ca
Cu
Fe
Hg
K
µm

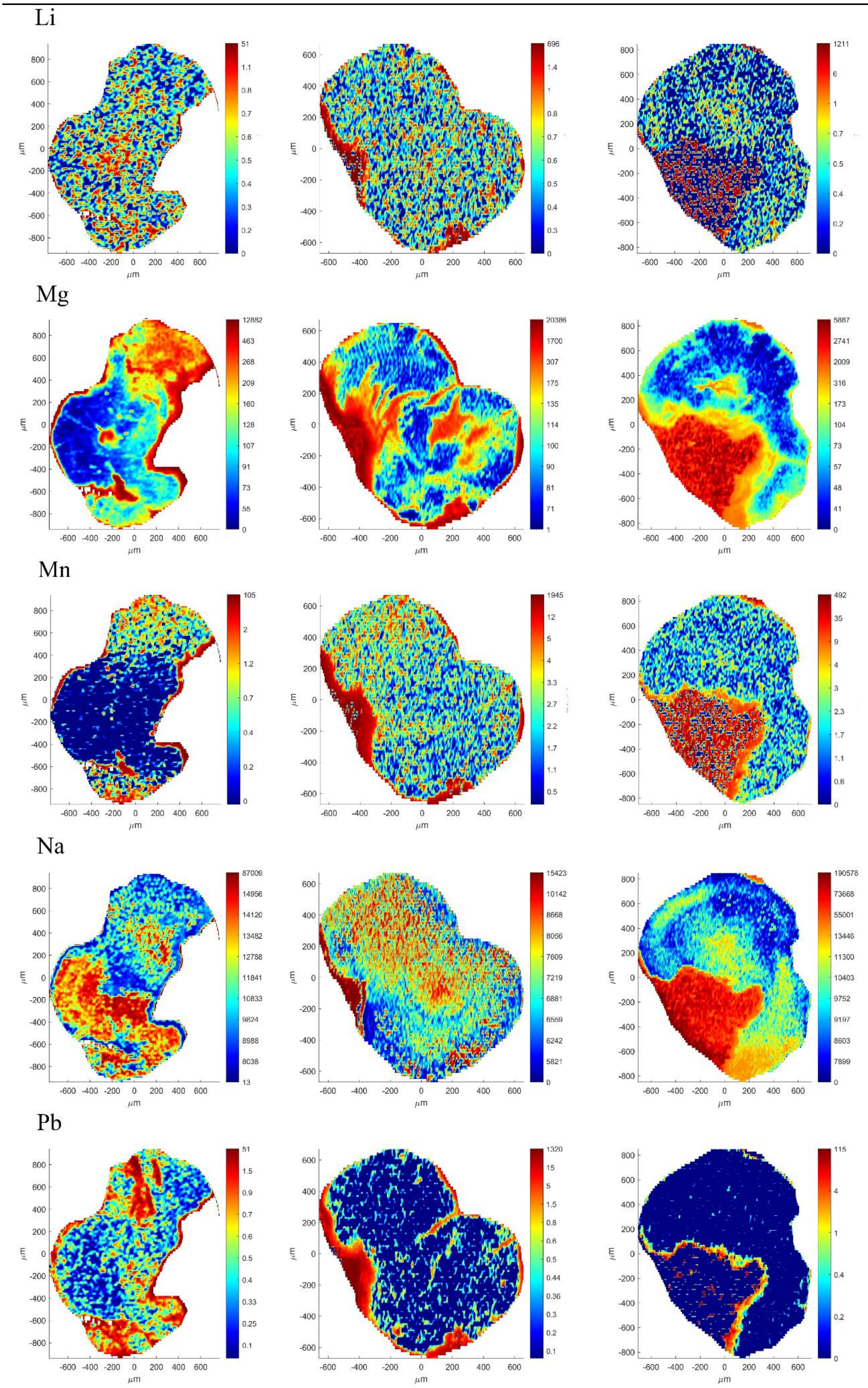
Li
Mg
Mn
Na
Pb
μm

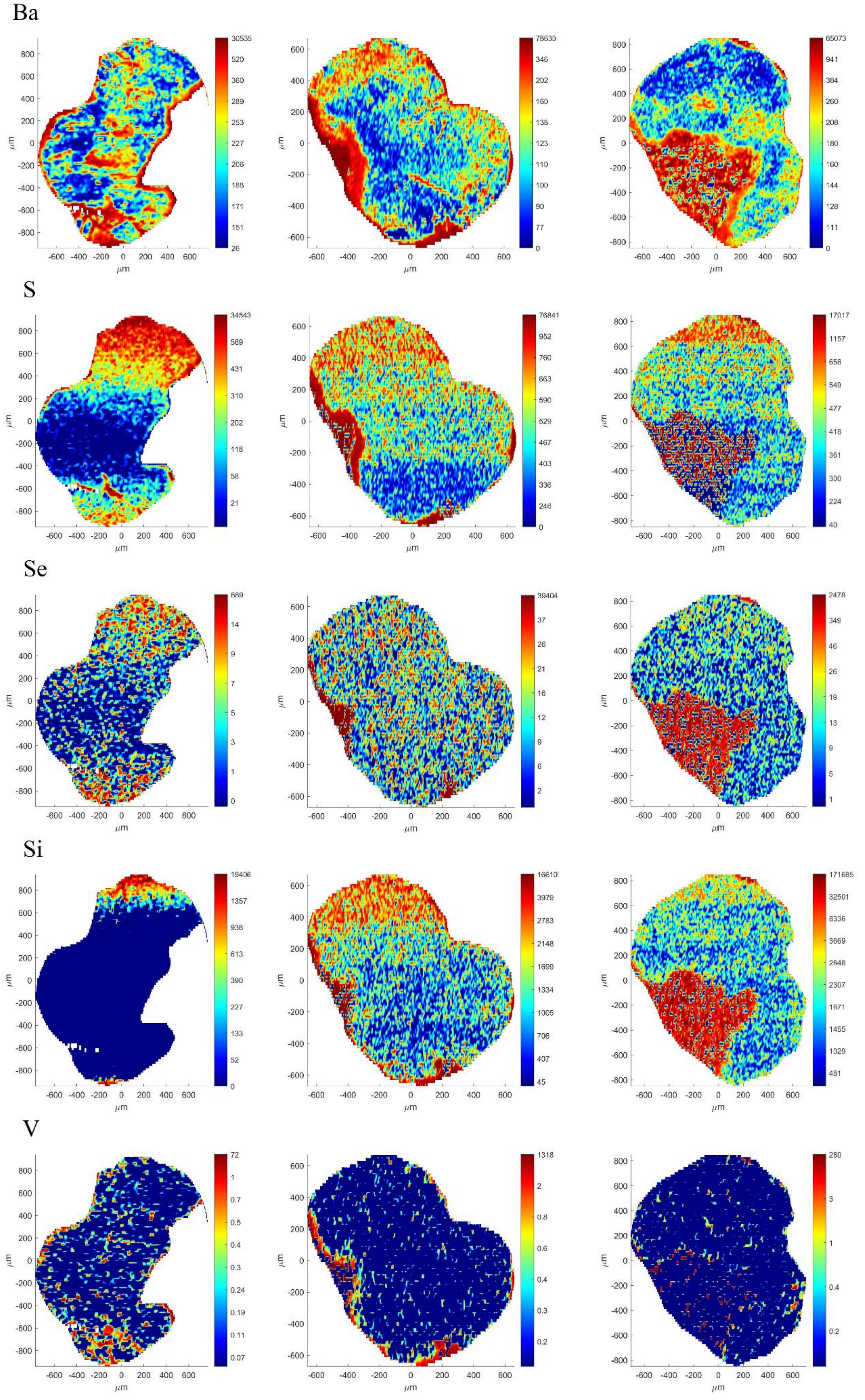
Ba
S
Se
Si
V

animals

MDPI

Zn

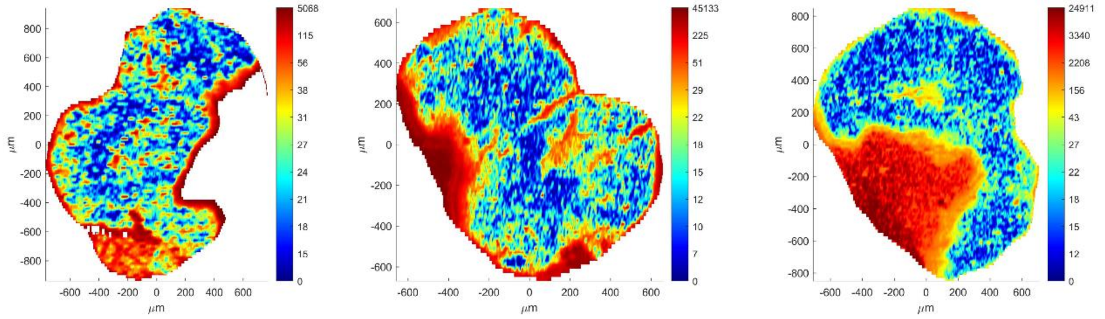

Figure S2. Microchemical map of elements in otolith, *s. grahami* sampled from the chishui river in march 2022 (a) Upper reaches (b) Middle reaches (c) Lower reaches.

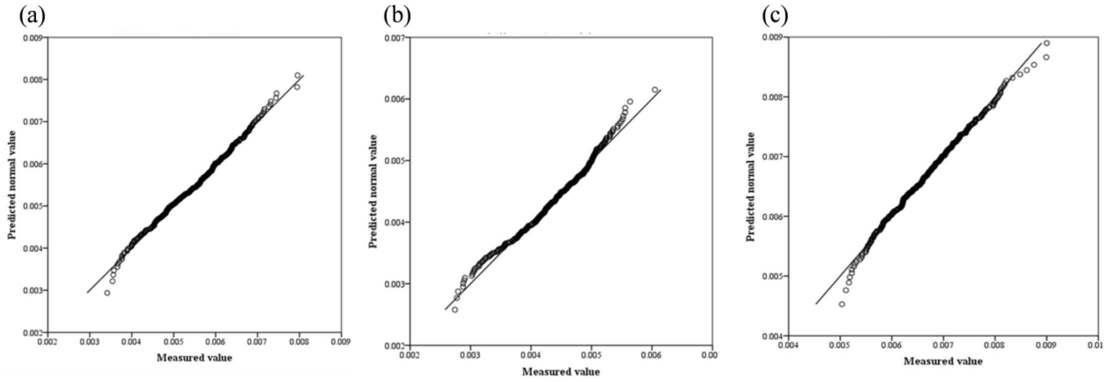

Figure S3. Normal Q-Q diagrams for the three groups. (a) Upper reaches (b) Middle reaches (c) Lower reaches.

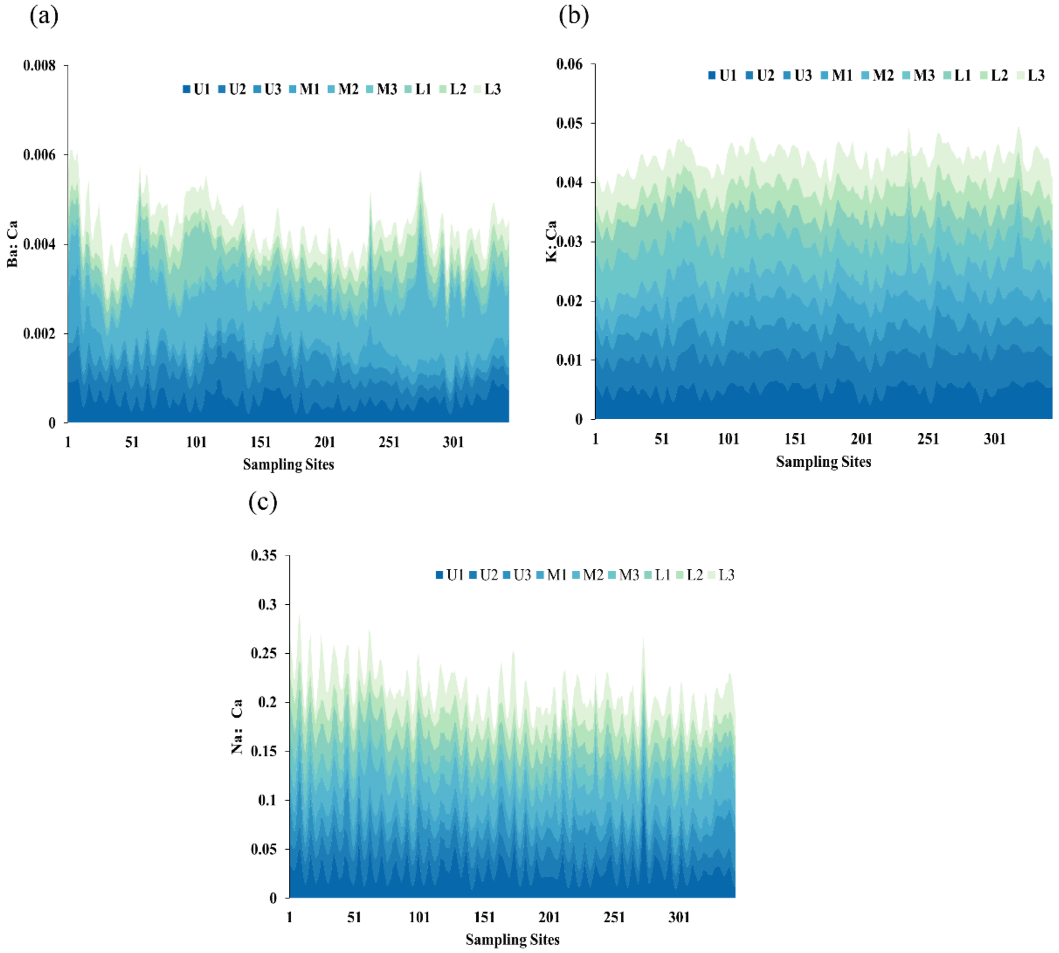

Figure S4. Point-fold line chart of the ratio of 3 elements (Ba, K, Na) to Ca of *S. grahami* sampled from the chishui river in march 2022. U1-U3 represents three samples from the Upper reach. M1-M3 represent three samples from the Middle reach. L1-L3 represent three samples from the Lower reach.

Table S1. One-sample K-S test for three reaches, s. grahami sampled from the chishui river in march 2022.

		**Upper reaches**	**Middle reaches**	**Lower reaches**
Number of cases		344	344	344
Normal parameters	Mean	0.005	0.004	0.007
	SD	0.000887	0.000614	0.000751
Most extreme difference	Absolute	0.042	0.044	0.031
	Positive	0.029	0.031	0.031
	Negative	-0.042	-0.044	-0.018
Test statistics		0.042	0.044	0.031
Asymptotic significance (two-tailed)		0.200	0.195	0.200

Table S2. Water sampling sites information table from the chishui river in march 2022.

Sampling Sites	Site Number	Sample Type	Longitude	Latitude	Fish Sample Numbers	Total Length Ranges of fish (mm)	Weight Ranges of Fish (g)	Habitat characteristics
YuDong	a	Fish and water	105.02984721	27.62433687	34	135-212	39.8-339.4	Spawning Ground
LuoDian	b	Water	105.02810372	27.68455467	-	-	-	None
Dawan	c	Water	105.08456914	27.71067921	-	-	-	None
YuTian	d	Fish and water	105.11162233	27.70224508	3	85-166	15.3-122.6	None
FaDianchang	e	Fish and water	105.17990975	27.75284062	2	172-183	91.2-113.4	Spawning Ground
ErLongqiangbao	f	Fish and water	105.17789108	27.75533058	25	121-247	23.5-383.6	Spawning Ground
ShiKan	g	Fish and water	105.15574575	27.77730395	3	128-166	42.5-98.3	None
BoLidaqiao	h	Water	105.20260132	27.74419169	-	-	-	Spawning Ground
LongDong	i	Fish and water	105.21059462	27.68740271	17	136-212	26.7-328.5	None
TianShengqiao	j	Water	105.20444418	27.65257678	-	-	-	Spawning Ground
BanJiujing	k	Fish and water	105.21680412	27.75649761	13	113-238	23.2-208.4	None
XinTan	l	Water	105.25115818	27.73237683	-	-	-	Spawning Ground
ChaHe	m	Water	105.30273264	27.71064276	-	-	-	None
BaMaoba	n	Water	105.25821531	27.79319500	-	-	-	Spawning Ground

Note: The delineation of potential spawning and natal origin areas (used for interpreting otolith core chemistry) was established based on long-term field monitoring data collected by our research team over the past 15 years in the Chishui River system. These areas were identified using consistent ecological criteria: (1) Direct observation of spawning aggregation and reproductive behavior during the breeding season; (2) Consistent records of high densities of eggs and early-stage larvae; and (3) Specific habitat characteristics known to favor reproduction of *S. grahami* (e.g., gravel or sandy substrate, specific water depth, and moderate flow velocity). As there is no prior published literature precisely mapping the spawning grounds in this specific river section, this long-term empirical dataset served as the foundation for our geographic grouping.

Table S3. Sites information table of measured sampling from the chishui river in march 2022.

Reaches	Sampling Sites Number	Measured sampling Number	Total Length (mm)	Weight (g)
Upper	a	U1	195	301.2
	a	U2	205	298.3
	a	U3	158	188.2
Middle	f	M1	233	353.2
	f	M2	192	283.5
	f	M3	225	331.2
Lower	i	L1	126	38.6
	k	L2	187	213.5
	k	L3	183	223.4
